# Inhibition and Biochemical Characterization of Methicillin-Resistant *Staphylococcus aureus* Shikimate Dehydrogenase: An *in Silico* and Kinetic Study

**DOI:** 10.3390/molecules19044491

**Published:** 2014-04-10

**Authors:** Claudia Avitia-Domínguez, Erick Sierra-Campos, José Manuel Salas-Pacheco, Hugo Nájera, Arturo Rojo-Domínguez, Jorge Cisneros-Martínez, Alfredo Téllez-Valencia

**Affiliations:** 1 Facultad de Medicina y Nutrición, Universidad Juárez del Estado de Durango, Av. Universidad y Fanny Anitúa S/N, Durango, Durango, C.P. 34000, Mexico; 2 Facultad de Ciencias Químicas, Universidad Juárez del Estado de Durango, Av. Artículo 123 S/N Fracc. Filadelfia, Gómez Palacio, Durango, CP. 35010, Mexico; 3 Instituto de Investigación Científica, Universidad Juárez del Estado de Durango, Av. Universidad S/N., Durango, Durango, C.P. 34000, Mexico; 4 Departamento de Ciencias Naturales, Universidad Autónoma Metropolitana, Unidad Cuajimalpa. Av. Vasco de Quiroga 4871, Colonia Santa Fe Cuajimalpa, Delegación Cuajimalpa de Morelos, Distrito Federal, C.P. 05300, Mexico

**Keywords:** MRSA, shikimate dehydrogenase, homology modeling, virtual screening, flexible docking, enzyme kinetics

## Abstract

Methicillin-resistant *Staphylococcus auerus* (MRSA) strains are having a major impact worldwide, and due to their resistance to all β-lactams, an urgent need for new drugs is emerging. In this regard, the shikimate pathway is considered to be one of the metabolic features of bacteria and is absent in humans. Therefore enzymes involved in this route, such as shikimate dehydrogenase (SDH), are considered excellent targets for discovery of novel antibacterial drugs. In this study, the SDH from MRSA (SaSDH) was characterized. The results showed that the enzyme is a monomer with a molecular weight of 29 kDa, an optimum temperature of 65 °C, and a maximal pH range of 9–11 for its activity. Kinetic studies revealed that SDH showed Michaelis-Menten kinetics toward both substrates (shikimate and NADP^+^). Initial velocity analysis suggested that SaSDH catalysis followed a sequential random mechanism. Additionally, a tridimensional model of SaSDH was obtained by homology modeling and validated. Through virtual screening three inhibitors of SaSDH were found (compounds **238**, **766** and **894**) and their inhibition constants and mechanism were obtained. Flexible docking studies revealed that these molecules make interactions with catalytic residues. The data of this study could serve as starting point in the search of new chemotherapeutic agents against MRSA.

## 1. Introduction

Nosocomial infections occur around the world at rates as high as 40% and are thus a serious health problem [1]. Among the bacteria, *Staphylococcus aureus* strains are one of the important causative agents of nosocomial infections of the blood stream, lower respiratory tract, skin and soft tissue [2]. Picao *et al.* reported on the prevalence of methicillin-resistant *S. aureus* (MRSA) in Latin America between 1997 and 2006 as part of the SENTRY study [3]. They found that more than one-third of *S. aureus* isolates (37.3%) were MRSA, significantly increased their prevalence from 33.8% in 1997 to 40.2% in 2006. Recently, Garza-González and Dowzicky [4] suggested MRSA numbers in Latin America has remained relatively stable between 2004 (44.6%) and 2010 (40.1%). *S. aureus* has also been notoriously developing antibiotic resistance, creating a serious problem for successful control of these infections. The effectiveness of vancomycin, which was once regarded as a drug of last resort to treat MRSA infections, has been marginalized by the emergence of vancomycin-resistant strains [5]. Moreover, *S. aureus* resistance to newer-generation drugs such as linezolid and daptomycin has also now been reported [6,7]. This creates an urgent need for new therapeutic agents to treat MRSA infections. In this regard, one approach is to look for small molecules that inhibit vital enzymes for bacteria survival; which may serve as a guide in the generation of new drugs.

In this perspective, a fundamental metabolic route in bacteria is the so-called “shikimate pathway”. This route, that combines glucose and pentose phosphate metabolism, involves seven reactions that convert erytrose-4-phosphate and phosphoenolpyruvate to chorismate, which is the precursor for the synthesis of important metabolites such as aromatic amino acids, ubiquinone and folate [8]. The shikimate pathway is present in plants, fungi, apicomplexan parasites and bacteria, but is absent in humans, a characteristic that makes its enzymes excellent targets for drug discovery [8,9,10].

Shikimate 5-dehydrogenase (SDH), the fourth enzyme in the shikimate pathway, catalyzes the NADPH-NADP^+^ dependent interconversion between dehydroshikimate (DSHK) and shikimate (SHK) [8]. Structurally, the enzyme may exist in monomeric [11,12] or dimeric [13,14,15] forms, with an average molecular weight of 29 kDa per monomer. The general structure comprises a N-terminal domain that binds with the substrate and a C-terminal NADP^+^ binding domain, that present the typical Rossmann fold of other nucleotide binding enzymes. According to the crystal structures reported in the Protein Data Bank, SDH presents two different conformations, an open and a closed, and the former is considered as the catalytic [16].

Nowadays, computer-assisted drug design tools such as homology modeling and virtual screening are powerful methodologies to find new enzyme inhibitors, and leads the way in the search of new antimicrobial agents [17]. In this context, the present study reported for the first time a new set of inhibitors of shikimate 5-dehydrogenase through a combination of studies involving cloning and biochemical characterization of shikimate 5-dehydrogenase from methicillin-resistant *S. aureus* (SaSDH), enzyme kinetics and different computational techniques.

## 2. Results and Discussion

### 2.1. Biochemical Characterization of SaSDH

**Figure 1:**
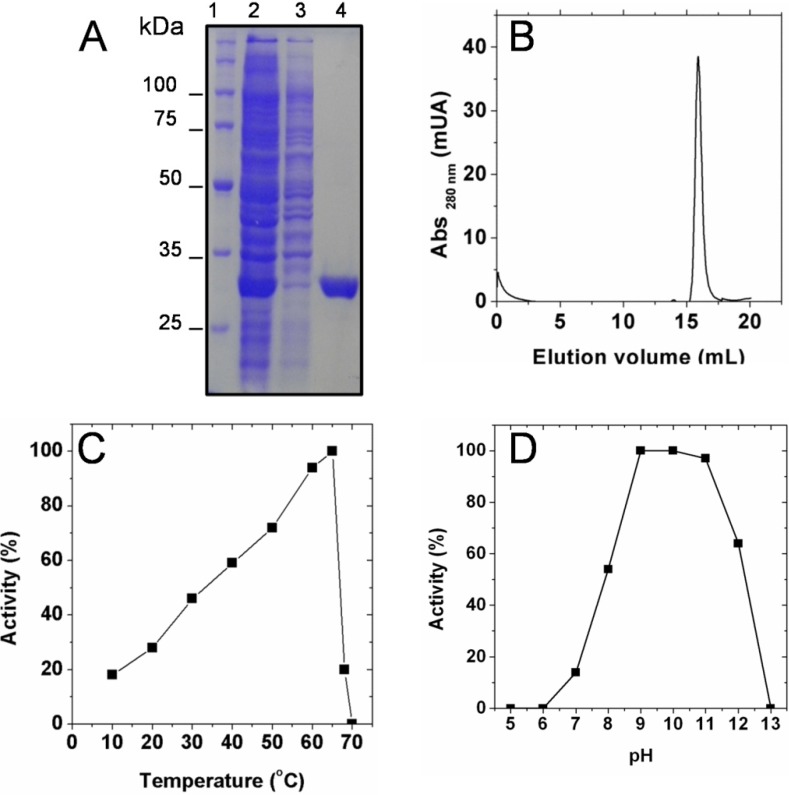
(**A**) Purification of SaSDH: lane 1 shows molecular weight markers; lane 2 shows the supernatant loaded into the column; lane 3 shows the proteins that did not bind to the resin; and lane 4 shows SaSDH eluted with 300 mM imidazole. (**B**) Elution profile of SaSDH under native conditions; the peak corresponds to a molecular weight of 29 kDa. (**C**) Temperature dependence of SaSDH activity. (**D**) pH dependence of SaSDH activity. The data are the mean values obtained from three independent experiments.

After purification to homogeneity (Figure 1A), the oligomeric state of SaSDH was determined using native size-exclusion chromatography; the chromatogram showed a single peak corresponding to a molecular weight of 29 kDa (Figure 1B). These results are in agreement with the predicted weight of the amino acid sequence of SaSDH [18] and the migration of the protein through SDS-PAGE gels (Figure 1A). This indicated that SaSDH exists as a monomer and is similar to its closest homologous *Staphylococcus epidermidis* SDH (SeSDH) [11].

On the other hand, activity measured at different temperatures, showed that the enzyme reached its maximum activity at 65 °C (Figure 1C), which is similar to that reported for *H. pylori* SDH (60 °C) [12]. Nevertheless *S. aureus* and *H. pylori* are mesophiles, *H. pylori* SDH retained above 80% of its activity at 70 °C, while SaSDH activity dropped to zero at the same temperature (Figure 1C). On the contrary, optimum temperature from the thermophile archaeon *A. fulgidus* SDH was around 90 °C, in fact, the activity of this enzyme was assayed at 87 °C [19].

The pH studies revealed that this enzyme exhibited a sustained highest activity over a pH range of 9–11 (Figure 1D), which is different from other bacterial SDHs, like those from *S. epidermidis* [11] and *M. tuberculosis* [20] which recorded a maximal activity at a pH of 11.0; *H. pylori* SDH [12] presented its optimum activity at pH of 9.0; while in SDH from *A. fulgidus* [18] and *C. glutamicum* [21] was at 7.0 and 7.53, respectively. These data suggested that catalysis in SaSDH depended on acid-basic amino acids.

### 2.2. Kinetic Constants and Reaction Mechanism

SDH catalyzes bisubstrate reactions, and assignment of inhibitor mechanism can be enhanced by understanding the kinetics mechanism of enzyme. A single enzymatic mechanism for dehydrogenation of shikimate has not been established. A number of kinetic studies point to a sequential mechanism [22,23], the data are consistent with an ordered bi-bi kinetic mechanism, where SDH binds first with 3-dehydroshikimate followed by NADPH binding to the enzyme catalytic site. However, the study of this enzyme may be difficult because the inhibition of plant SDH by dehydroshikimate produces non-linear Dixon plots, particularly at low shikimate concentrations, indicating dead end complex formation [24,25].

**Figure 2:**
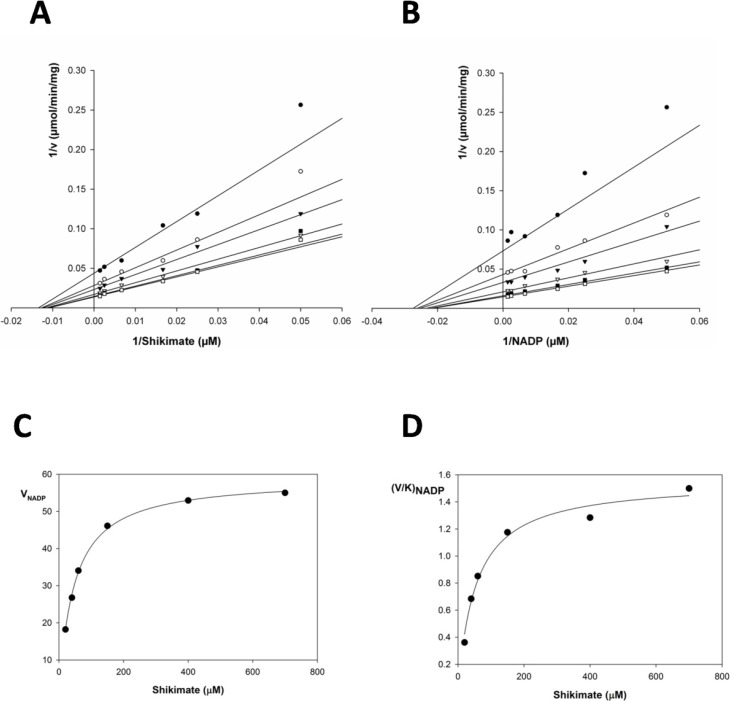
(**A**, **B**) Double reciprocal analysis of initial velocities under conditions where either NADP^+^ or shikimate is varied at different fixed concentrations of the co-substrate. (**C**, **D**) Secondary plot of slope of B vs shikimate concentrations.

In order to examine whether a ping-pong or a sequential mechanism is operative in SaSDH, NADPH formation was measured as a function of shikimate at different fixed concentrations of NADP^+^ and vice-versa. Figure 2 shows all kinetic data and secondary plots for titration of SaSDH, experiments performed to determine the kinetic mechanism of the NADP^+^ linked reaction were complicated and during initial attempts to carry out a full titration analysis, it became apparent that titrations using shikimate concentrations spanning between 20–100 μM were yielding seemingly parallel lines in double reciprocal plots. Only by spanning concentrations of this substrate over (150–700 μM) it was possible to clearly identify the points of reciprocal plot intersection and the apparent increase in slope was more pronounced when sub-saturating concentrations of NADP^+^ (400–700 μM), which suggested an inhibition by shikimate (Figure 2A). Moreover, under conditions where the concentrations of NADP^+^ was varied, the slopes exhibited an uncompetitive relationship at low concentration of shikimate (20–100 μM) but appeared to increase subtly with increasing fixed shikimate concentrations (150–700 μM), suggesting that substrate inhibition by NADP^+^ also occurs (Figure 2B). Despite this problem, the double reciprocal plots of 1/v *versus* 1/shikimate at various fixed NADP^+^ concentrations gave a family of straight lines with different slopes and intercepts which intersected at a common point in the third (lower left-hand) quadrant of the plot (Figure 2A, B). This is typical of a mechanism that involves reaction of the enzyme with both substrates before the release of any products, rather than a ping-pong mechanism, involving a distinct modified enzyme. This result suggested that dehydrogenation of shikimate proceeded through a sequential bi-bi reaction mechanism, with the formation of a ternary NADP^+^-SDH-SHK complex. However, no clear distinction between a rapid equilibrium random bi-bi system or a steady-state ordered bi-bi mechanism can be made based solely on the initial velocity patterns [26]. Hence, secondary replots of *V_NADP_* with shikimate concentrations and *(V/K)_NADP_* with shikimate showed that data fitted to the hyperbolic pattern, which corresponded to a random sequential mechanism (Figure 2C, D). These data suggested that SaSDH catalyzed reverse reaction follows a random bi-bi mechanism.

Due to these results, the kinetic data were re-evaluated by a global non-linear regression analysis using computer simulation program SigmaPlot v12.3. Data were fitted to several models of bi-substrate reactions, all of which were analyzed using SigmaPlot Enzyme Kinetics Module v1.3. Akaike Information criterion corrections (AICc) for sample size values were used to determine the best fitting equation following SigmaPlot instructions, along with standard errors of the parameters (*K_m_* and *V_max_*) estimates [27]. The best equation had the lowest AICc value, with a minimum of 7 AICc units different from the next lowest. The initial velocity kinetics suggested a random sequential reaction mechanism, but this sort of analysis cannot be considered conclusive, and needs to be verified with product inhibition studies, and until this is done, it is important to report both dissociation constants. Table 1 summarizes the values of the kinetic constants determined by global and secondary plot data fitting to the equation derived for random bi-bi system. Global fitting of the data to equation 1 yielded a *V_max_* for SHK and NADP^+^, at saturating levels of both substrates, of 233 and 251 µmol/min/mg, respectively; values of *K_cat_* = 135 for shikimate and 146 s^−1^ for NADP^+^, were obtained. Furthermore, the dissociation constant (*K_ia_*) for both substrates were calculated, giving values of 21.22 μM for SHK and 21.0 μM for NADP^+^. These were used to derive the turnover number (*K_cat_*) and *K_cat_/K_m_*. The results showed in Table 1 are in fair agreement with previously published data for other bacterial SDHs [11,12,16,18,19,28].

**Table 1:** Kinetic parameters for SaSDH.

Substrate	*K_m_* (μM)	*V_max_* (μmol/min/mg)	*K_cat_* (s^−1^)	*K_cat_/K_m_* (M^−1^x s^−1^)	*K_ia_*	*K_ia_/K_m_*
Shikimate	37.46 ± 3.7	233.6 ± 1.5	135.81	3.62 × 10^6^	21.224	1.76
NADP^+^	42.55 ± 9.9	251.41 ± 7.2	146.16	3.43 × 10^6^	21.015	2.02

### 2.3. SaSDH Inhibition

The quest for small molecules that specifically inhibit SDH function has proven to be particularly challenging. The inhibitors reported to date for bacterial SDHs are hydrophobic compounds with complex structures that inhibit SDH activity competitively and noncompetitively with respect to shikimate [12]. Thus, it is important to find other types of SDH inhibitors with different structures, which might serve as a starting point for maximizing specificity. To this end, we looked for inhibitors of SaSDH through a virtual screening strategy.

#### 2.3.1. Homology Modeling and Virtual SCREENING

**Figure 3:**
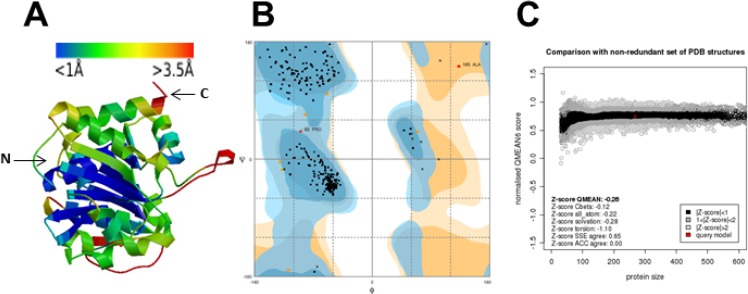
(**A**) 3D model of SaSDH in ribbons colored by per-residue inaccuracy (the N- and C- terminals are indicated with arrows); the upper bar shows the corresponding deviation according to the color. The SHK binding site is located in the blue region. (**B**) Ramachandran plot obtained from model protein geometry evaluation (dark blue, light blue, and orange correspond to the favored, allowed and outlier regions respectively). (**C**) Normalized QMEAN6 score graphic showing the position (red cross) of the model in the set of PDB structures used for the evaluation and the Z-score value.

In order to obtain structural information on SaSDH and for virtual screening from a small molecules database, a three-dimensional model of the enzyme was generated (Figure 3A), as described in the Materials and Methods section. The stereochemical quality of the model was revisited and validated by four different programs. According to Errat2 [29], the model obtained an overall quality factor of 94.33 (upper 85 means a good quality); in RAMPAGE [30] only 0.8% of the residues (Pro62 and Ala85) were in the outlier region in Ramachandran plot, 2.2% were in the allowed region and 97% in the favored region of the plot (Figure 3B). Similar results were obtained from Molprobity [31] evaluation, but here only Ala85 was outlier. Q-MEAN [32,33] reported a normalized QMEAN6 score of 0.75 (close to 1 is the ideal) and a Z-score of −0.26 (close to zero is the ideal) (Figure 3C). Additionally, SaSDH model colored by per-residue inaccuracy [32,33] showed that the domain selected for virtual screening had a deviation less than 1Å from the structures used for evaluation (Figure 3A). Structural analysis of the model revealed that the SaSDH has the typical α/β folding of other SDHs, showing in the N-terminal region the SHK binding site and the Rossmman domain for NADP^+^ binding in the C-terminal. Because the model was constructed using as template the crystal structure of SeSDH with open conformation, SaSDH model also presented the same folding, in fact, structural alignment between SaSDH model and SeSDH (3DON) reported an rmsd (root mean square deviation) of 0.077 Å. Taking together, all the data supported that the SaSDH 3D model is of high quality and could be used for virtual screening.

Once the model was built, a rigid body docking procedure for virtual screening with a library of small molecules was performed. Because the enzyme is absent in humans, we selected the substrate (SHK) binding site as the target for virtual screening (Figure 3A). From the one thousand small molecules studied, a database of 38,355 poses was generated. The one hundred molecules with the highest binding energies were selected, and the capacities of these molecules to inhibit SaSDH were assessed. Inhibition assays using a concentration of 200 µM of the latter molecules were performed. Table 2 shows the inhibition percent of the SaSDH activity induced by the ten most active compounds; some of the molecular properties of these compounds are also described.

The three most potent molecules found through virtual screening, 6-hydroxy-2,3-dihydrobenzo[b]furan-3-one (compound **894**), 7-hydroxy-2,2,8-trimethyl-2,3-dihydro-4*H*-chromen-4-one (compound **766**), and 2,2'-bithiophene-5-carboxylic acid (compound **238**), were selected for further kinetic and structural studies.

**Table 2:** Molecular properties and drug likeness of the ten most potent. SaSDH inhibitors found through virtual screening.

Compound Structure	Molecular Weight ^a^	H-bond Donor ^a^	H-bond Acceptor ^a^	LogP ^a^	Drug Likeness ^a, b^	Binding energy kcal/mol	% Inhibition 200 µM
**894** 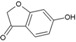	150.1	1	3	0.97	0.13	−11.93	99
**766** 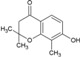	206.2	1	3	2.43	−0.76	−12.55	87
**238** 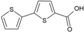	210.3	1	4	3.32	−099	−12.38	87
**626** 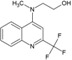	270.2	1	2	2.83	−0.40	−12.85	49
**463** 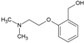	195.3	1	3	1.05	0.24	−11.89	45
**62** 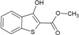	208.2	1	4	2.69	−0.70	−11.98	33
**291** 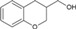	164.2	1	2	1.66	−0.80	−12.27	31
**692** 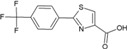	273.2	1	4	3.87	−0.99	−11.90	31
**306** 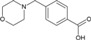	221.2	1	4	1.23	0.20	−11.90	31
**637** 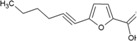	192.2	1	3	2.83	−0.79	−12.25	29

^a^ Obtained from molsoft prediction server [34]; ^b^ Values between −1 and 2 indicate drug-like molecules, values closer to 2 are better.

#### 2.3.2. Kinetic Study of the SaSDH-Inhibitor Complex

Although it might seem easy to compare the potency of this set of inhibitors acting on the same target, but in practice, it was not so straightforward. There is more than one way to report the inhibitory capacity of compounds, the inhibition constant, *K_i_* and the *i_0.5_* (the 50% inhibition concentration) value. Usually biochemical investigations often determine the *K_i_*, but rarely determine an *i_0.5_*. The *K_i_* is determined by means of simple linear regression applied to Dixon plot, while that *i_0.5_* is obtained by constructing a dose-response curve. Other aspect important is that SDH catalyzes bisubstrate reaction, and assignment of an inhibitor mechanism can be achieved by understanding the kinetic mechanism of enzyme. Therefore, we decided to use the method reported by Cortés *et al.* which permitted us to calculate both parameters simultaneously [35].

**Figure 4:**
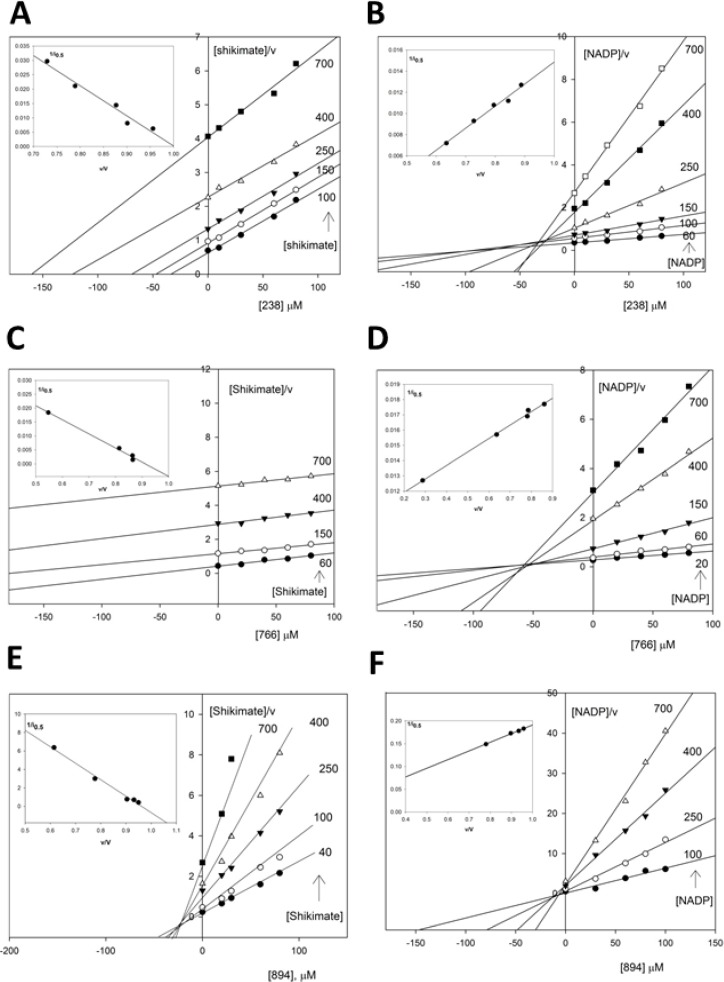
SaSDH inhibition patterns by compounds **238** (**A** and **B**), **766** (**C** and **D**) and **894** (**E** and **F**). A Cornish-Bowden plot was drawn using the data obtained from the kinetic studies in order to confirm the inhibition pattern and the inhibition constants (*K_ic_* and *K_iu_*) were determined by interpretation of the *1/i_0.5_ versus v/V* (insets). *V* and *v* represent the maximal velocity and the velocity in the absence and presence of the inhibitor with a given concentration of the substrate, respectively.

In order to study the type of inhibition of compounds on SaSDH, pharmacological and kinetic parameters (*i_0.5_*, *K_ic_* and *K_iu_*) were determined at various concentrations of these compounds, using SHK and NADP^+^ respectively, as substrates. In Figure 4, the plots of reciprocal rate multiplied by the substrate concentrations (*S/v*) *versus* inhibitor concentration in the presence of SHK and NADP^+^ are shown. The intercept of the extrapolated line on the [i] axis, that corresponds to each compound concentration, is *‒i_0.5_*. This provided a single and accurate way of estimating *i_0.5_*, which is important if it is to be used to calculate the inhibition constant [35].

In order to further explore the molecular mechanism of action of these compounds, the kinetic parameters of inhibition was measured. The Cornish-Bowden plots for the inhibited reactions showed that the compounds **238** and **766** exhibited the characteristics of competitive inhibition with SHK (Figure 4A, C), whilst the **894** is a mixed-competitive inhibitor with respect to SHK and mixed-uncompetitive with respect to NADP^+^ (Figure 4E, F). These findings were confirmed by secondary plots for the three compounds. When **238** and **766** were assessed with NADP^+^ as the variable substrate, the Cornish-Bowden plots indicated that both compounds exhibited characteristics of uncompetitive inhibition (Figure 4B, D). The mixed inhibition exhibited by **894** is a pattern usually observed in multisubstrate enzyme catalyzed reactions. However, the uncompetitive inhibition shown by **238** and **766** indicated that there is no reversible link between the inhibitor and the NADP^+^.

The *i_0.5_* values obtained for compounds **238**, **766** and **894** were 122.94, 343.4 and 142.9 μM respectively. Since the results from the inhibition assays for 400 µM of either substrates demonstrated that these compounds displayed moderate inhibition against SaSDH, compared with other inhibitors reported for bacterial SDH [12]. If this value is considered, we may mistakenly dismiss these compounds, because the *i_0.5_* value depends on concentrations of the enzyme (or target molecule), the inhibitor, and the substrate along with other environmental conditions such as pH, ionic strength and temperature. Therefore, an accurate determination of the *K_i_* value is required, which has an intrinsic thermodynamic quantity that is independent of the substrate but dependent on the enzyme and inhibitor.

According to this, the dissociation constant, *K_i_*, for compounds **238**, **766** and **894** was determined. It has long been common practice to determine the inhibition constants by use of Dixon plots. However, the usefulness of the Dixon plot is limited by the fact that it cannot be applied to uncompetitive inhibition. Therefore, the Cornish-Bowden plot was used to obtain the *K_i_* value for the binding of the inhibitor of enzyme-substrate complex and demonstrated the mechanism of inhibition of these small molecules. The compounds **238** and **766** behaved according to a competitive mode of inhibition (They affected *K_m_* value for shikimate), and these inhibitors had a better affinity for the free enzyme than for the enzyme-substrate complex, being their *K_ic_* < *K_iu_*, while that the compound **766** had a *K_ic_* = 19.7 for shikimate and a *K_iu_* = 11.3 for NADP^+^ (Table 3). In contrast, the compound **894** showed values of *K_ic_* = 900.9 µM and *K_iu_* = 5.2 µM for NADP^+^. These results demonstrated the importance of determining the values of both parameters for each inhibitor and agreed with those previously reported [36].

**Table 3:** Inhibition kinetics of compounds **238**, **766** and **894** against SaSDH.

Substrate	Inhibitor	*K_ic_* (μM)	*K_iu_* (μM)	*i_0.5_* at 400 μM of substrate	Pattern
Shikimate	238	9.53	NA	122.94	Competitive
NADP^+^	238	ND	48.3	107.52	Uncompetitive
Shikimate	766	19.76	NA	343.43	Competitive
NADP^+^	766	ND	11.35	564.2	Uncompetitive
Shikimate	894	58.3	1470	142.9	Mixed-competitive
NADP^+^	894	900.9	5.2	614	Mixed-uncompetitive

NA, not applicable. ND, not determined. *K_ic_* is the dissociation rate constant for inhibitor binding to the substrate site. *K_iu_* represents the dissociation constant for inhibitor binding to the enzyme-substrate complex.

#### 2.3.3 Flexible Docking of the SaSDH-Inhibitor Complex

**Figure 5:**
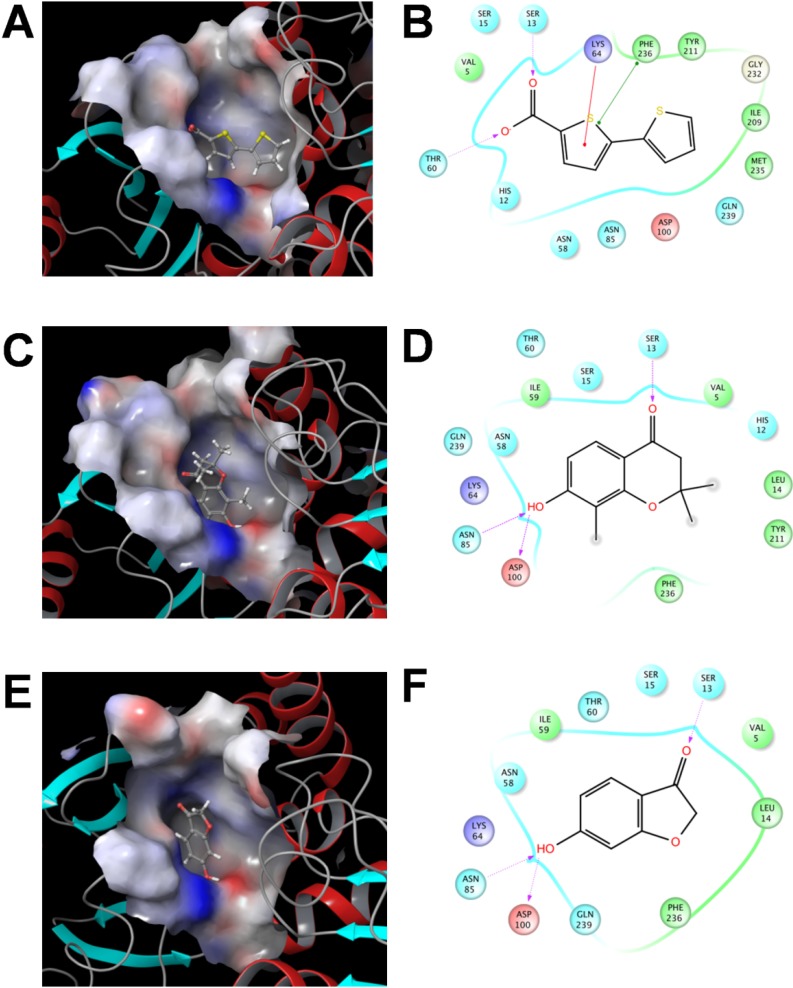
2D and 3D plots showing binding of compounds **238**, **766** and **894** (**A**, **C** and **E**, respectively) to the SaSDH active site (showing electrostatic surface; neutral residues are depicted in white, while negative and positive charged residues are colored in red and blue respectively), showing interactions between the enzyme and compounds **238**, **766** and **894** (**B**, **D** and **F**, respectively). Dashed lines with arrow heads indicate H-bond formation; single dots indicate cation-pi interactions, and dots in both extremes of the line indicate pi-pi interactions.

To obtain more detailed structural information on enzyme-inhibitor interactions, an induced fit docking procedure (flexible docking), was applied to these three molecules towards the binding pocket of the modelled protein (Figure 5A, C, E). Both, side chains of amino acids in the binding site and the inhibitor showed flexibility. On the contrary, neither protein nor ligands are flexible in the rigid body docking procedure, flexibility of the ligand is explored through conformer generation. The binding energies of the best complex for each inhibitor were −4.53, −4.82, and −6.0 kcal/mol for compound **238**, **766**, and **894**, respectively. These energies cannot be compared with those reported in the rigid body docking procedure (Table 2), because both were obtained under different conditions using distinct software. The docking poses showed that compound **238** formed hydrogen bonds with Ser13 and Thr60, a cation-pi interaction with Lys64, and a pi-pi interaction with Phe236 (Figure 5B). Compounds **766** and **894** participated in hydrogen bonds with Ser13, Asn85 and Asp100 (Figure 5D, F). Structurally, the three molecules share some similarities with SHK. Compounds **766** and **894** each have a carbonyl group and a six-carbon ring with a hydroxyl group, which are responsible for the interactions described above, while compound **238** has a carboxyl group that forms the hydrogen bond with Ser13. According to these results, it seems that in the SeSDH-SHK crystallographic complex [11], the interactions of the compounds with Ser13, Lys64, Asn85, and Asp100 are central to the desired inhibition mechanisms.

To date, only Han *et al.* found through high throughput screening strategy inhibitors of *H. pylori* SDH, these compounds were the cucurmin, two chromene derivatives, and maesaquinone diacetate [12]. Structurally there is no similarity between these molecules and the ones reported in this study, in fact, the unique common characteristic is the presence of carboxyl groups as substituent. Additionally, compounds **238**, **766** and **894** are considerably smaller, therefore, these SaSDH inhibitors represent a new chemical scaffold that can serve as a guide to design more potent and selective inhibitors. Furthermore, the drug-likeness model scores estimated for these inhibitors (Table 2) validate the consideration of these compounds as drug-like molecules.

## 3. Experimental

### 3.1. SaSDH Gene Cloning

Genomic DNA from the ATCC MRSA252 strain (purchased from ATCC^®^, Manassas, VA, USA) was used for SaSDH gene (AroE) cloning via PCR. The oligonucleotides 5'GCCATATGAAA TTTGCAGTTATCGGAAATCC3' (forward) and 5'GCGGATCCTTATTCTCCTTTTAATTG3' (reverse) were used, in which the restriction sites for NdeI and BamHI, respectively, are underlined. The PCR product was cloned using PCR BLUNT II® TOPO® vector (Invitrogen, Carlsbad, CA, USA) and then sequenced. Subsequently, the DNA was subcloned into pET28a(+) to generate a protein with a 6His-tag at its N-terminus and finally introduced via transformation into *E. coli* BL21(DE3)pLysS cells (Novagen, Madison, WI, USA) for gene over-expression.

### 3.2. Enzyme Purification

Luria-Bertani medium (250 mL) containing 50 µg/mL kanamycin was inoculated with bacteria transformed with pET28a(+) vector containing the SaSDH gene. These bacteria were grown at 37 °C until the absorbance at 600 nm reached 0.5. At this time, over-expression was induced using 0.4 mM IPTG; after 3 h, the cells were harvested by centrifugation, and the resulting pellet was washed with 100 mM Tris-HCl, pH 8.0 (buffer A). The cells were suspended in buffer A added with the protease inhibitor PMSF (200 µM) and then lysed by sonication. SaSDH was purified by passing the supernatant through a Ni-NTA affinity column and washed with buffer A supplemented with increasing concentrations of imidazole, 20 mM (50 mL), 50 mM (50 mL), 100 mM (20 mL), and 200 mM (15 mL). SaSDH was eluted with buffer A plus 300 mM (10 mL) imidazole. The Bradford method was used to determine the protein concentration [37].

### 3.3. Molecular Weight Determination

Native size-exclusion chromatography was performed using a Superdex 200 10/300 GL column, and 75 µg of SaSDH was injected into the column. The molecular weight was determined using a calibration curve constructed using different proteins of various molecular weights, and fitting the data to the equation described elsewhere [38].

### 3.4. Enzyme Activity

Enzyme activity was measured spectrophotometrically at 25 °C at 340 nm, following NADPH generation (ε = 6.19 × 10^3^ M^−1^ cm^−1^). The reaction mixture contained buffer A plus 1 mM SHK, 1 mM NADP^+^, and 50 ng of SaSDH.

### 3.5. Reaction Mechanism and Kinetic Parameters

The kinetic parameters for shikimate and NADP^+^ were determined by independently varying the concentration of each from 0.01 to 1 mM while maintaining the concentration of the other at 1 mM. The data were linearized by the double-reciprocal transformation of Lineweaver and Burk [39]. Primary plots (1/v *versus* 1/NADP^+^) patterns were used to confirm the sequential reaction mechanism of SaSDH. The Km and dissociation constant for shikimate were determined from the secondary plots of y intercepts and slopes against 1/NADP^+^ plots. The inversion of the data matrix allowed the determination of the dissociation constant for NADP^+^.

Additionally, kinetic parameters *K_m_*, *V_max_*, and dissociation constant values for NADP^+^ and shikimate were also estimated by fitting each data set to the bisubstrate Michaelis-Menten model, by using an iterative nonlinear least-squares method (SigmaPlot EK module). Following the notation of Cleland [25], the bisubstrate equation for a bi-bi mechanism is:

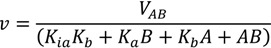
(1)
where *A* is the first binding substrate concentration, *B* is the second binding substrate constant of the *EA* binary complex, and *K_a_* and *K_b_* are the Michaelis constants for *A* and *B*. *K_ia_* values were obtained by considering alternatively NADP^+^ and shikimate as the first binding substrate in the fitting procedure. Finally, when substrate inhibition patterns were observed in a data set, the outlier observations were ignored for *K_m_* estimation.

### 3.6. Inhibition Assays

The data were fitted to equations for competitive, uncompetitive, non-competitive and mixed inhibition, respectively, using the kinetic module of SigmaPlot program v12.3. All experiments were repeated at least twice.

The 50% inhibition concentrations (*i_0.5_*) of compounds against SaSDH were determined and analyzed by fitting to [Equation (2)]. The inhibition mechanism and inhibition constant (*K_ic_* and *K_iu_*) were studied by fitting the inhibition data to [Equation (3)]:

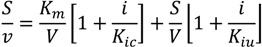
(2)

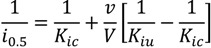
(3)

In these equations, *S* and *i* are the substrate (shikimate or NADP^+^) and inhibitor concentrations. *K_ic_* is the inhibition constant derived from the intercepts, whereas *K_iu_* is the constant derived from the slope of *1/i_0.5_* vs *v/V* plots.

### 3.7. Biochemical Determinations

The optimum temperature was calculated by measuring enzyme activity at temperatures that varied from 10 to 80 °C. The optimum pH was calculated by measuring the enzyme activity at a range of pH values from 5 to 12.8. The following different buffers were used to obtain the desired pH: citrate-phosphate (pH 5–7), tris-HCl (pH 8–9), glycine-sodium hydroxide (pH 9–10), sodium bicarbonate-sodium hydroxide (pH 10–11), and potassium chloride-sodium hydroxide (pH 12–12.8).

### 3.8. Homology Modeling

The SaSDH model was constructed using the *homology model* tool in the computational package MOE [40]. Because the crystal structure of SeSDH has been reported [11] and share a 70% amino acid sequence identity with SaSDH (the highest measurement obtained based on the PSI_BLAST [41] alignment obtained using the PredictProtein server [42]), using SeSDH structure as the template (PDB ID: 3DON).

Ten different intermediate models were constructed. These models were the result of permutational selection of side-chain rotamers and different loop candidates. The intermediate model with the best packing index according to the chosen scoring function was selected as the final model and was subjected to an energy minimization using the Amber 99 force field [43]. Finally, the stereochemical quality of the model was determined using the external validation software Errat2 [29], Rampage [30], Molprobity [31], and Q-Mean [32,33].

### 3.9. Virtual Screening

A core set of the Maybridge Ro3 Diversity Fragment Library [44], which comprises one thousand small molecules that represent the chemical diversity of the entire library, was used to find inhibitors of SaSDH through a rigid body docking procedure in MOE [40]. Their three-dimensional low-energy conformations and atomic partial charges were obtained using the Gasteiger-Marsilli algorithm [45], and energy minimization using the MMFF94x force field [46] were determined in MOE until a gradient of 0.05 kcal/mol was reached. Conformations from each molecule with energies higher than 3 kcal/mol were eliminated from the process to avoid internal strains. Assignment of atomic partial charges and energy minimization in MOE were applied to the SaSDH model for virtual screening. Because this enzyme is absent in humans, the substrate (SHK) binding site was used to search for potential inhibitors. The residues forming the docking site (Val5, Ser13, Ser15, Asn58, Ile59, Thr60, Lys64, Asn85, Asp100, Phe236 and Gln239) were determined by alignment to those for the SHK binding site in the SeSDH crystallographic complex (PDB ID: 3DOO) [11]. Approximately 80,000 random orientations, with variations in position and molecular rotation, were assessed per conformer of each ligand. The score for each of these was calculated using the London dG Scoring function in MOE, considering the spatial compatibility of the binding site, the internal energy of the ligand, desolvation energy of each atom, and protein–ligand interactions. A database with the binding energy for each conformer was obtained, and 100 molecules with the highest scores were selected for inhibition studies.

### 3.10. Induced Fit Docking (IFD)

This protocol was applied to the three most potent inhibitors using Schrödinger Software, suite 2013-3 [47]. The protein (SaSDH model generated in MOE), ligands, and substrate (SHK) were prepared using *Protein Preparation Wizard* [48], *LigPrep* v2.8 [49], *Epik* v2.6 [50], and *Prime* v3.4 [51] programs. *Glide* v6.1 [52] was used to prepare protein-ligand complexes, and *Induced Fit Docking* [53] was used for flexible docking studies. The grid was generated based on the SHK binding site, using the fault settings for Van der Waals radius and charge scaling. In *Glide*, standard precision and extra precision protocols were applied. The complexes obtained from the former were used for IFD. For each inhibitor, 10 complexes in IFD were generated; the complex with the best energy score was selected for structural analysis.

## 4. Conclusions

To the best of our knowledge, neither the characteristics of SaSDH, and as a consequence, nor the existence of inhibitors have been previously described. Furthermore, the inhibition mechanism of these compounds was different to the reported for *H. pylori* SDH inhibitors, besides their marked structural difference, making of these molecules a new chemical scaffold. Therefore, the data reported here may provide a starting point for the search for more potent inhibitors that could lead to the discovery of new chemotherapeutic agents against MRSA and other nosocomial bacteria.
